# The Effect of Mycotoxin Deoxynivalenol on Haematological and Biochemical Indicators and Histopathological Changes in Rainbow Trout (*Oncorhynchus mykiss*)

**DOI:** 10.1155/2014/310680

**Published:** 2014-03-04

**Authors:** Iveta Matejova, Helena Modra, Jana Blahova, Ales Franc, Petr Fictum, Marie Sevcikova, Zdenka Svobodova

**Affiliations:** ^1^Department of Veterinary Public Health and Welfare, Faculty of Veterinary Hygiene and Ecology, University of Veterinary and Pharmaceutical Sciences Brno, Palackeho tr. 1/3, 612 42 Brno, Czech Republic; ^2^Department of Pharmaceutics and Biopharmacy, Faculty of Pharmacy, University of Veterinary and Pharmaceutical Sciences Brno, Palackeho tr. 1/3, 612 42 Brno, Czech Republic; ^3^Department of Pathological Morphology and Parasitology, Faculty of Veterinary Medicine, University of Veterinary and Pharmaceutical Sciences Brno, Palackeho tr. 1/3, 612 42 Brno, Czech Republic

## Abstract

Deoxynivalenol (DON), produced by the *Fusarium *genus, is a major contaminant of cereal grains used in the production of fish feed. The effect of mycotoxin deoxynivalenol on rainbow trout (*Oncorhynchus mykiss*) was studied using a commercial feed with the addition of DON in a dose of 2 mg/kg feed. The fish (*n* = 40) were exposed to the mycotoxin for 23 days. The trout were divided into two groups, control and experimental groups. Control groups were fed a commercial feed naturally contaminated with a low concentration of DON (225 **μ**g/kg feed); experimental groups were fed a commercial feed with the addition of DON (1964 **μ**g/kg feed). Plasma biochemical and haematological indices, biometric parameters, and histopathological changes were assessed at the end of the experiment. The experimental groups showed significantly lower values in MCH (*P* < 0.05). In biochemical indices, after 23-day exposure, a significant decrease in glucose, cholesterol (*P* < 0.05), and ammonia (*P* < 0.01) was recorded in the experimental group compared to the control group. Our assessment showed no significant changes in biometric parameters. The histopathological examination revealed disorders in the caudal kidney of the exposed fish. The obtained data show the sensitivity of rainbow trout (*O. mykiss*) to deoxynivalenol.

## 1. Introduction

Mycotoxins are toxic secondary metabolites produced by different types of filamentous fungi. The most relevant mycotoxins to animal health and production are produced by *Aspergillus, Fusarium, *and* Penicillium *genera. Mycotoxins are thermally and chemically stable and this renders them resistant to feed manufacturing techniques [1].

Deoxynivalenol (DON), also known as vomitoxin, is a trichothecene mycotoxin produced by the *Fusarium* genus. Deoxynivalenol and zearalenone belong to the most prevalent mycotoxins produced by the *Fusarium* species [2]. Although DON is the least toxic type of trichothecene, it can cause significant harm to animals and humans [3].

Animal feed components and finished feedstuffs normally contain this mycotoxin, it is one of the most frequently found mycotoxin in cereal grains, such as wheat, barley, and corn [4].

Despite the fact that rainbow trout belongs to a carnivorous species, commercial salmonoid feeds contain plant components. Due to a decrease in the availability of fish meal for the production of aquaculture feeds, alternative protein sources need to be used in fish feed production. When we use more plant source ingredients in commercial feeds for farmed fish, we increase the possibility that mycotoxins contaminate those feeds [5].

Deoxynivalenol causes a broad variety of toxic effects in animals, the toxicity is well recognized in mammals. The main effect at the cellular level is the inhibition of protein synthesis through it being bound to the ribosomal subunit [6]. Chronic oral exposure induces anorexia, decreased weight gain, reduction in feed conversion, gastrointestinal hemorrhaging, inflammation, and immune system alterations. The effects of deoxynivalenol depend on dose and duration of exposure, age, species, health, and nutritional status. There are differences in sensitivity to DON contaminated feed in fish. Rainbow trout (*O. mykiss*) is extremely sensitive [7].

## 2. Materials and Methods

### 2.1. Animals

The experiment was carried out on one-year-old rainbow trout (*O. mykiss*) obtained from a commercial fish farm. Groups of ten fish were randomly distributed into four tanks of 200 L volume with dechlorinated tap water. The test was performed using a flow-through system with the bath exchanged every 12 h and individually aerated. A photoperiod regime of 12 h light : 12 h dark was used. Trout were acclimated for two weeks and during this acclimation were supplied twice a day with commercial pellets (BioMar, Denmark) at a total rate of 1% of body weight.

### 2.2. Experimental Diet

The control fish were fed a commercial diet (BioMar, Denmark) containing rapeseed oil, blood meal, fish meal, soya cake, sunflower cake, rapeseed meal, horse beans, wheat, soya concentrate, fish oil, pea proteins, vitamins, and minerals.

The experimental diet was prepared by adding DON to commercial pellets in several separate steps. The amount of 32.50 g of Eudragit E (Basic Butylated Methacrylate Copolymer) was dissolved in 227.50 g of acetone on the electromagnetic stirrer for a period of 60 minutes (solution A). 60 mL of this solution was put aside (solution B). Into each of the three vials containing 5 mg of DON, 10 mL from the solution B was injected for the reconstitution of DON. Next, the dissolved content of these vials was added to the original solution A. The vials were then rinsed with the rest of solution B (10 mL for each vial) and solutions (A and B) were mixed together. The resultant common solution was divided into the two equal parts with the weight of 130 g that is equivalent to 7.5 mg of DON.

2470.75 g of pellets and 13.00 g of AEROSIL were added to a cubic blender and mixed for 5 minutes at 40 rpm (“Blend A”). 130 g of solution with a content of 7.5 mg of DON was uniformly and carefully poured onto the surface of mixed excipients and this moistened mixture was kneaded for 5 minutes at 40 rpm. The same procedure was performed with the “Blend B”. The final mixtures were placed in a hot air dryer and dried at 50°C for 4 hours.

The polymer forms a specific layer on the surface of the pellets, which is formulated from the solid dispersion of the active ingredient fixed in a polymer. As a result, it is assumed that there is a highly uniform content of active substance in each of the individual pellets.

### 2.3. Analysis of Mycotoxin

The contents of deoxynivalenol, 3-acetyldeoxynivalenol, 15-acetyldeoxynivalenol, diacetoxyscirpenol, fumonisin B_1_ and B_2_, HT-2 toxin, T-2 toxin, nivalenol, ochratoxin A, and zearalenone in control and experimental feed were analyzed by liquid chromatography-tandem mass spectrometry (LC-MS/MS) by Metrology and Testing Laboratory (Institute of Chemical Technology, Prague). The analyzed mycotoxin contents are described in Table 1.

### 2.4. Experimental Protocol

Fourty fish were divided into control (*n* = 20) and experimental (*n* = 20) groups. Control groups were twice a day fed the commercial diet used during the initial acclimation period at a total rate of 1% of body weight. Experimental groups were fed with mycotoxin-contaminated feed at a dose of 2 mg/kg feed. The samples were taken after 23 days from the beginning of the experiment. Water temperature during the test ranged from 14.0 to 15.1°C, pH ranged from 7.9 to 8.2, and oxygen saturation of the water ranged from 80.5 to 95.2%. The physicochemical parameters of the water used in the test during the experiment were total ammonia 0.1–0.5 mg/L, NO_3_^−^ 20–30 mg/L, NO_2_^−^ 0.1–0.4 mg/L, and Cl^−^ 20–25 mg/L.

### 2.5. Haematology Profile

Blood samples were taken from each fish by puncturing the caudal vessel and stabilized with sodium heparin (50 IU per 1 mL of blood). Heparinized blood samples were used for the evaluation of haematological indicators including erythrocyte count (RBC), haemoglobin concentration (Hb), hematocrit (PCV), mean erythrocyte volume (MCV), mean erythrocyte haemoglobin (MCH), mean corpuscular haemoglobin concentration (MCHC), and leukocyte count (WBC). Samples were determined according to Svobodova et al. [8].

### 2.6. Biochemical Profile

For biochemical analysis, a part of heparinized blood, after centrifugation at 855 g for 10 min at cooled centrifuge (4°C), was used. Biochemical parameters including albumins (ALB), total proteins (TP), glucose (GLU), ammonia (NH_3_), triacylglycerols (TRIG), lactate (LACT), cholesterol (CHOL), alkaline phosphatase (ALP), alanine aminotransferase (ALT), aspartate aminotransferase (AST), lactate dehydrogenase (LDH), calcium (Ca^2+^), and inorganic phosphate (PHOS). Plasma biochemical indicators were measured using a biochemical automatic analyzer Konelab 20i (ThermoScientific, Czech Republic) and commercial test kits (BioVendor, Czech Republic).

### 2.7. Biometric Parameters

After blood sampling, the fish were stunned with a blow to the head and killed by spinal transection. Then, the biometrical indices were defined (total and standard length, body and liver weight) from which there were derived and calculated somatic parameters such as the Fultons condition factor and the hepatosomatic index. The Fultons condition factor (CF) was calculated using the formula CF = (body weight [g]/standard length [cm^3^]) × 100. The hepatosomatic index (HSI) was calculated using the following formula: HSI = liver weight/body weight × 100.

### 2.8. Histological Examination

The samples of gills, skin, liver, cranial, and caudal kidney and spleen of ten fish from each group were immediately fixed in buffered 10% neutral formalin. The samples were later dehydrated, embedded in paraffin wax, sectioned on a microtome at a thickness of 4 *μ*m, and stained with haematoxylin and eosin (H + E). The sections were examined by light microscopy and photographed using a digital camera.

### 2.9. Statistical Analysis

The results of the haematological and biochemical examinations and biometric parameters were carried out with UNISTAT statistica 5.6 software. Data were first tested for normality (Shapiro-Wilk test). When necessary, logarithmic transformations were used for the analysis of variance. A one-way analysis of variance (ANOVA) and a Tukey-HSD testwere applied. If the normal distribution was not satisfied, a nonparametric Kruskal-Wallis test was applied. Significance was accepted at *P* < 0.05.

## 3. Results

### 3.1. Haematology Profile

The results of the analyses of the haematological indices of both control and experimental groups after 23 days from the beginning of the experiment are presented in Table 2. The exposure caused a significant decrease of MCH (*P* < 0.05) in the experimental fish. The other measured haematological indices showed no statistically significant differences between the experimental and control groups.

### 3.2. Biochemical Profile

The results of plasma biochemical indicators are presented in Table 3. The experimental rainbow trout exposed to the feed with deoxynivalenol showed statistically significant lower values of glucose, cholesterol (*P* < 0.01), and ammonia (*P* < 0.05) in comparison with negative control*.*

### 3.3. Biometric Parameters

The mean values of fish total and standard body length, body and liver weight, Fultons condition factor, and hepatosomatic index did not show significant differences (Table 4). 

### 3.4. Histopathological Examination

**Figure 1:**
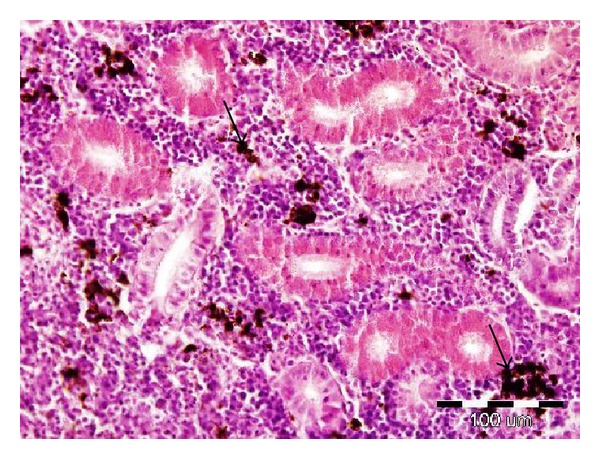
Effect of deoxynivalenol exposure on caudal kidney histology. Hyaline droplet degeneration of tubular epithelial cells (arrows). HE, 400x.

The histopathological examination revealed severe hyaline droplet degeneration in the tubular epithelial cells (tubulonephrosis) of the caudal kidney in 9 out of 10 fish fed the diet containing DON (Figure 1). No substantial histopathological changes were demonstrated in the other tissues (gills, skin, liver, cranial kidney, and spleen).

## 4. Discussion

The study showed posttreatment changes in the haematological and biochemical profiles and histopathological changes in rainbow trout fed a commercial feed with the addition of the mycotoxin deoxynivalenol. No fish mortality was observed in the control or experimental groups during the test.

**Figure 2:**
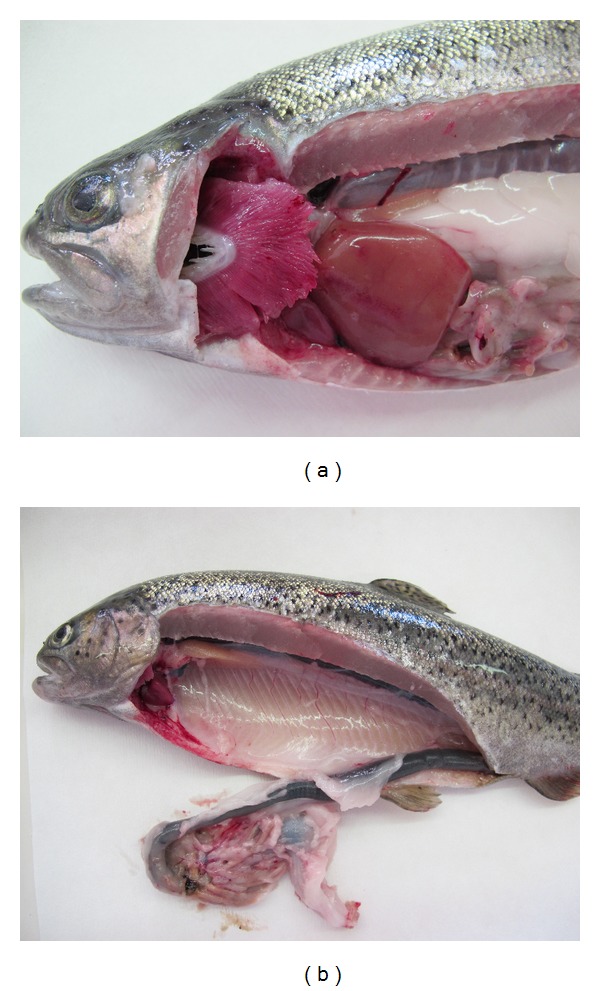
Experimental rainbow trout after exposure of DON-haemorrhages in the liver and gastrointestinal tract.

The main haematological response of rainbow trout after 23 days exposure to DON was a statistically significant decrease in MCH (*P* < 0.05). The lower values of MCH, MCHC, MCV, and Hb suggested that the concentration of haemoglobin in red blood cells is lower due to an anaemic condition [9]. The common clinical symptoms of DON toxicity include among others gastrointestinal hemorrhaging which we demonstrated in our study (Figure 2) and might be the reason for the anaemic condition of the trout. A moderate increase in the erythrocyte count might be caused by the higher percentage of immature red blood cells in the circulation and might be the other reason for the MCH, MCHC, and MCV decrease in the present study [10].

The main biochemical response of rainbow trout to the effect of DON showed a decrease in glucose, cholesterol (*P* < 0.05), and ammonia (*P* < 0.01) in comparison with the control groups. The lower values of these parameters might be caused by a lower intensity of metabolism. Here the effect of feeding a diet containing DON on feed intake and fish weight was observed. The fish weight was nonsignificantly lower in the DON-treated group. The decrease in feed intake could subsequently lead to a decreased intensity in nitrogen metabolism [11]. The decrease in blood glucose during the 23 days exposure can be attributed to the high utilization of glucose for hypoxic conditions and oxidation [12]. It is well established that DON consumption inhibits protein synthesis [6]. In the current study, feed with the addition of toxin caused a nonsignificant decrease in the total protein in the plasma when compared with the control fish.

We observed severe hyaline droplet degeneration in the tubular epithelial cells of the renal tubules of the caudal kidney in the experimental group. The kidney is a target organ of certain toxicants; it is a major route for the excretion of foreign chemicals. On the other hand, Hooft et al. reported that deoxynivalenol caused considerable morphological changes in the liver, including subcapsular haemorrhages, subcapsular edema, altered hepatocytes, and fatty infiltration [7]. In the current study, we have demonstrated subcapsular haemorrhages in the liver in some of the fish fed diets containing the DON (Figure 2).

In conclusion, the results of the present study indicate that exposure of deoxynivalenol in a dose of 2 mg/kg feed induces significant changes in the haematological and biochemical parameters and in the histopathological examination of rainbow trout. The alterations of these parameters may provide a better understanding of the toxicological effect of mycotoxin deoxynivalenol on aquaculture fish, such as rainbow trout.

## Figures and Tables

**Table 1:** Mycotoxin concentration in control and experimental feed.

Mycotoxin contamination *μ*g/kg	Control feed	Experimental feed
Deoxynivalenol	225	1964
3-Acetyldeoxynivalenol	ND^1^	ND
15-Acetyldeoxynivalenol	ND	ND
Diacetoxyscirpenol	ND	ND
Fumonisin B_1_	ND	ND
Fumonisin B_2_	ND	ND
HT-2 toxin	ND	ND
T-2 toxin	ND	ND
Nivalenol	ND	ND
Ochratoxin A	ND	ND
Zearalenone	ND	1

^1^ND: not detectable.

**Table 2:** Values of haematological indices 23 days after the beginning of the experiment.

Indices	Control mean ± SD (*n* = 20)	Experimental mean ± SD (*n* = 20)
RBC (T·L^−1^)	1.41 ± 0.36	1.58 ± 0.41
Hb (g·L^−1^)	76.94 ± 12.78	69.49 ± 12.92
PCV (L·L^−1^)	0.30 ± 0.06	0.29 ± 0.05
MCV (fL)	228.14 ± 66.62	198.26 ± 53.05
MCH (pg)	57.86 ± 16.26	46.93 ± 14.12*
MCHC (g·L^−1^)	250.31 ± 27.58	235.67 ± 22.97
WBC (G·L^−1^)	12.13 ± 4.97	14.23 ± 3.58

Significant difference between test groups (*P < 0.05).

RBC: erythrocyte count, Hb: haemoglobin concentration, PCV: haematocrit, MCV: mean erythrocyte volume, MCH: mean erythrocyte haemoglobin, MCHC: mean corpuscular haemoglobin concentration, WBC: leukocyte count.

**Table 3:** Values of biochemical parameters 23 days after the beginning of the experiment.

Indices	Control mean ± SD (*n* = 20)	Experimental mean ± SD (*n* = 20)
ALB (g·L^−l^)	15.69 ± 2.90	15.25 ± 2.74
TP (g·L^−l^)	37.45 ± 5.77	36.38 ± 3.98
GLU (mmol·L^−1^)	4.84 ± 0.79	4.36 ± 0.48*
NH_3_ (*μ*mol·L^−1^)	398.14 ± 75.85	280.79 ± 57.99**
TRIG (mmol·L^−1^)	1.90 ± 0.40	1.63 ± 0.68
LACT (mmol·L^−1^)	2.62 ± 0.98	2.10 ± 0.69
CHOL (mmol·L^−1^)	6.50 ± 1.43	5.54 ± 1.12*
ALP (*μ*kat·L^−1^)	1.64 ± 0.78	1.40 ± 0.69
ALT (*μ*kat·L^−1^)	0.33 ± 0.12	0.42 ± 0.28
AST (*μ*kat·L^−1^)	7.66 ± 2.35	7.71 ± 1.85
LDH-L (*μ*kat·L^−1^)	16.57 ± 6.50	16.77 ± 6.81
Ca^2+^ (mmol·L^−1^)	2.32 ± 0.15	2.32 ± 0.16
PHOS (mmol·L^−1^)	3.49 ± 0.41	3.67 ± 0.42

Significant difference between test groups (*P < 0.05*;* ^∗∗^ *P* < 0.01).

ALB: albumins, TP: total proteins, GLU: glucose concentration, NH_3_: ammonia, TRIG: triacylglycerols, LACT: lactate, CHOL: cholesterol, ALP: alkaline phosphatase, ALT: alanine aminotransferase, AST: aspartate aminotransferase, LDH: lactate dehydrogenase, Ca^2+^: calcium, PHOS: inorganic phosphate.

**Table 4:** Biometrical indices 23 days after the beginning of the experiment.

Indice	Control mean ± SD (*n* = 20)	Experimental mean ± SD (*n* = 20)
Total length	26.10 ± 1.37	25.89 ± 0.85
Standard length	23.44 ± 1.25	23.67 ± 0.85
Body weight	199.33 ± 33.22	185.48 ± 29.95
Liver weight	2.98 ± 0.69	2.92 ± 0.66
Fultons condition factor	1.54 ± 0.17	1.39 ± 0.17
Hepatosomatic index	1.49 ± 0.26	1.58 ± 0.33
